# Ferroptosis Altered microRNAs Expression in HT-1080 Fibrosarcoma Cells Based on Small RNA Sequencing and Bioinformatics Analysis

**DOI:** 10.3390/nu16060873

**Published:** 2024-03-17

**Authors:** Qian Zhang, Qiwen Wang, Haoxuan Ding, Caihong Hu, Jie Feng

**Affiliations:** Key Laboratory of Animal Nutrition and Feed of Zhejiang Province, College of Animal Sciences, Zhejiang University, Hangzhou 310058, China

**Keywords:** ferroptosis, HT-1080 cells, small RNA sequencing, microRNAs, regulatory network

## Abstract

Iron is an essential trace element in the human body. However, excess iron is harmful and may cause ferroptosis. The expression and role of microRNAs (miRNAs) in ferroptosis remain largely unknown. A model of ferroptosis induced by ferric ammonium citrate in HT-1080 cells was established in this study. The miRNAs expression profiles of the control and iron groups were obtained using small RNA sequencing and verified using qRT-PCR. A total of 1346 known miRNAs and 80 novel miRNAs were identified, including 12 up-regulated differentially expressed miRNAs (DE-miRNAs) and 16 down-regulated DE-miRNAs. SP1 was the most important upstream transcription factor regulating DE-miRNAs. The downstream target genes of DE-miRNAs were predicted based on miRDB, TargetScan, and miRBase databases, and 403 common target genes were screened. GO annotation and KEGG analysis revealed that the target genes were mainly involved in various biological processes and regulatory pathways, especially the MAPK signaling pathway and PI3K-Akt signaling pathway. Afterwards, a target genes network was constructed using STRING and Cytoscape, and the hub genes were compared with the ferroptosis database (FerrDb V2) to discover the hub genes related to ferroptosis. *EGFR*, *GSK3B*, *PARP1*, *VCP*, and *SNCA* were screened out. Furthermore, a DE-miRNAs-target genes network was constructed to explore key DE-miRNAs. hsa-miR-200c-3p, hsa-miR-26b-5p, and hsa-miR-7-5p were filtered out. Comprehensive bioinformatics analysis of miRNAs and its upstream and downstream regulation in ferroptosis in HT-1080 cells using small RNA sequencing is helpful for understanding the role of miRNAs in iron overload-related diseases and ferroptosis-targeted therapy for cancer.

## 1. Introduction

Iron is an integral microelement required for all life forms and the most abundant transition metal in the human body, which functions in numerous biological processes such as energy metabolism, oxygen transport, and DNA biosynthesis [1,2]. However, dysregulation of iron homeostasis triggers the development of a wide range of diseases, and iron-mediated cytotoxicity has been regarded as a major cause of tissue and organ damage [3]. At a physiological level, excess free iron may cause oxidative damage or even lead to cell death [4]. Ferroptosis is a unique modality of regulated cell death distinct from apoptosis [5], including unique pathological, biochemical, and immunological features associated with increased iron accumulation, lipid peroxidation, and reactive oxygen species (ROS) [6]. Ferroptosis plays a key role in a variety of pathophysiological conditions including cancer [7]. Cancer development is based on specific mutations in oncogenes related to the redox system, and cancer cells are more prone to iron accumulation, which makes them more susceptible to ferroptosis than normal cells [8].

**Figure 1:**
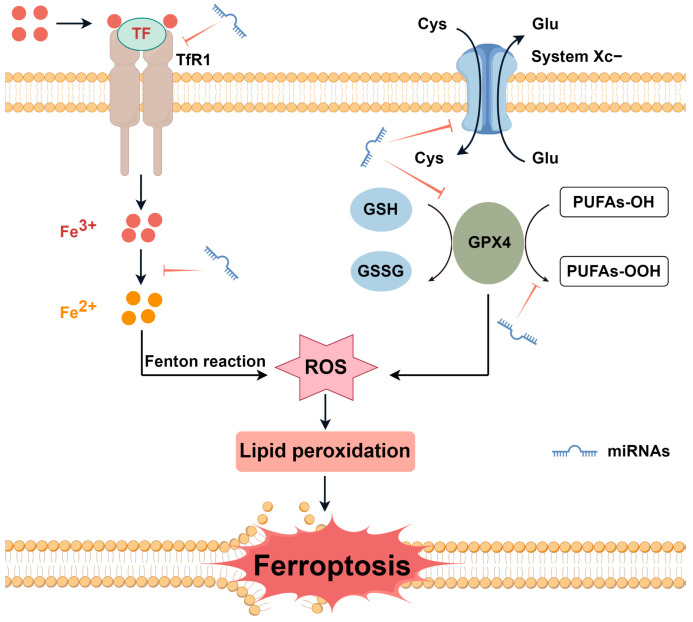
The mechanisms of iron and miRNAs regulating ferroptosis. Ferroptosis is an iron-dependent non-apoptotic form of cell death and is regulated by iron metabolism, lipid metabolism, glutathione-GPX4 pathway, glutamate/cystine transport, and other processes. miRNAs influence ferroptosis by regulating the above processes. Cys—cystine; Glu—glutamate; GPX4—glutathione peroxidase 4; GSH—glutathione; GSSG—glutathione disulfide; miRNAs—microRNAs; PUFAs—polyunsaturated fatty acids; ROS—reactive oxygen species; TF—transferrin; and TfR1—transferrin receptor 1. By Figdraw.

Noncoding RNAs are ubiquitous in transcription within mammalian genomes and constitute the majority of the transcribed genome, with only 1–2% encoding proteins [9]. MicroRNAs (miRNAs) are a class of small noncoding RNAs, which play a vital role in regulating a wide range of biological processes [10]. miRNAs can inhibit mRNA translation or promote mRNA degradation, known as endogenous physiological regulators of gene expression [11]. miRNAs regulate the expression of more than 30% of genes in the body, and their functions are closely related to cell proliferation, apoptosis, tissue and organ formation, and the occurrence of various diseases including cancer [12]. There is a close relationship between iron, miRNAs, and ferroptosis (Figure 1). The regulatory mechanisms of ferroptosis involve oxidation system and antioxidant system, including iron metabolism, lipid metabolism, glutathione-GPX4 pathway, and glutamate/cystine transport [13]. An increasing body of evidence reveals the critical role of miRNAs in ferroptosis by targeting various ferroptosis players mentioned above [14]. Meanwhile, miRNAs can post-transcriptionally regulate the expression of genes related to iron acquisition, export, storage, utilization, and regulation of systemic iron homeostasis [15]. In addition, iron plays a key role in miRNAs processing as a functional component of heme [16].

HT-1080 fibrosarcoma cells exhibit a robust ferroptotic response and are frequently utilized as a model system to investigate the mechanisms underlying ferroptosis [17]. In our previous study, we confirmed that ferric ammonium citrate (FAC) induced intracellular iron overload, leading to ferroptosis in HT-1080 cells [18]. However, our comprehension of the impact of ferroptosis on miRNAs expression remains limited. To fill this knowledge gap, herein, we established a ferroptosis model in HT-1080 cells induced by FAC and used small RNA sequencing to thoroughly investigate the role played by miRNAs. We elucidated the expression profile of miRNAs in ferroptosis in HT-1080 cells and further demonstrated its potential metabolic and signaling pathways based on bioinformatics analysis. This could provide new perspectives at the transcriptional level for identifying biomarkers and therapeutic approaches for iron overload-related diseases.

## 2. Materials and Methods

### 2.1. Cell Lines and Cell Culture

The HT-1080 cell line used in this study was obtained from American Type Culture Collection. Cells were cultured in Dulbecco’s Modified Eagle Medium/Nutrient Mixture F-12 (DMEM/F-12, Gibco, Carlsbad, CA, USA), supplemented with 10% fetal bovine serum (FBS, Gibco, Carlsbad, CA, USA) and 1% penicillin/streptomycin (Pen-Strep, Sigma, St. Louis, MO, USA) at 37 °C in a 5% CO_2_ atmosphere. Upon reaching 80%–90% confluence, cells were passaged at a ratio of 1:3.

### 2.2. Construction of Ferroptosis Model in HT-1080 Cells

The method of constructing the ferroptosis model in HT-1080 cells has been previously described [18]. HT-1080 cells were seeded at a density of 1 × 10^4^ cells/well in 96-cell plates (Corning, Corning, NY, USA) so that each well was allowed to reach 80% confluence, and treated with FAC (Sigma, St. Louis, MO, USA) at 0, 2, 4, 6, and 8 mM for 24 h. Then, cell viability was measured using Cell Counting Kit-8 assay (CCK-8, Biosharp, Heifei, China) according to the manufacturer’s instructions. To visualize the characteristic cell morphology, the cells were seeded at a density of 2 × 10^5^ cells/well in 6-well plates (Corning, Corning, NY, USA). After reaching 80% confluence, cells were treated with different concentrations of FAC for 24 h. Then, an inverted microscope (Leica DCF295, Wetzlar, Germany) was used to observe cell morphology.

### 2.3. Total RNA Extraction

To harvest RNA samples, HT-1080 cells treated in the 6-well plates were collected after 24 h. Considering the sample requirements for sequencing, cells treated with a concentration of 4 mM FAC were selected. Total RNA in control group (CO) and iron group (IO) was extracted using TRIzol™ Reagent (Invitrogen, Carlsbad, CA, USA). RNA degradation and contamination were checked using 1% agarose gels. RNA purity and concentration were measured using NanoDrop 2000 (Thermo Fisher Scientific, Wilmington, DE, USA). RNA integrity was mentored using Agilent 2100 Bioanalyzer (Agilent Technologies, Santa Clara, CA, USA).

### 2.4. Small RNA Sequencing

The small RNA sequencing was conducted by a commercial company (Novogene Bioinformatic Technology, Beijing, China). The control group and the iron group each consisted of three duplicates. The miRNA was isolated using miRNeasy Mini Kit (QIAGEN, Dusseldorf, Germany) according to the manufacturer’s instructions. The concentration and quality of the samples were evaluated using NanoPhotometer spectrophotometer (IMPLEN, Munich, Germany). After the sample’s quality met the sequencing quality requirement, miRNA libraries were generated using NEBNext Multiplex Small RNA Library Prep Set for Illumina (NEB, Ipswich, MA, USA) following the manufacturer’s recommendations. Subsequently, the index coding samples were clustered on the cBot Cluster Generation System using TruSeq SR Cluster Kit v3-cBot-HS (Illumina). Finally, the library preparations were sequenced on the Illumina Hiseq 2000 platform. Then, processed reads of length ranging from 18 to 35 nt were mapped to their reference genome and analyzed using Bowtie [18]. Finally, the sequences were aligned with the specified range sequences in miRbase to obtain matches for known miRNAs in each sample. Meanwhile, miREvo [19] and miRDeep2 [20] software were integrated to predict novel miRNAs.

### 2.5. Differential Expression of miRNAs

Differential expression analysis between the control group and the iron group was performed using the DESeq package (version 1.24.0) in R (version 4.2.3). A corrected *p*-value < 0.05 was considered indicative of differentially expressed miRNAs (DE-miRNAs).

### 2.6. Prediction of Transcription Factors (TFs) and Target Genes

The TFs of DE-miRNAs were predicted using FunRich (version 3.1.4). DE-miRNAs were subjected to FunRich, and the top 10 TFs were exported. The mirDIP platform (version 5.3.0.1, database version 5.2.3.1, https://ophid.utoronto.ca/mirDIP/download.jsp/, accessed on 1 November 2023) was utilized for predicting target genes, selecting the miRDB, miR-base, and TargetScan databases, with the score classification set to very high (1%). Venn analysis was used to screen the shared target genes from the three databases for subsequent analysis, and the Venn diagram was plotted using the VennDiagram package (version 1.7.3) in R (version 4.2.3).

### 2.7. Functional Annotation and Pathway Enrichment

Gene ontology (GO) and Kyoto Encyclopedia of Genes and Genomes (KEGG) enrichment analyses were performed with target genes using the clusterProfiler package (version 4.6.2) and enrichplot package (version 1.18.4) in R (version 4.2.3). *p*-value < 0.05 and q-value < 0.05 were considered statistically significant in GO and KEGG enrichment analyses.

### 2.8. Protein–Protein Interaction (PPI) Network Establishment and Analysis of Hub Genes

PPI network for DE-miRNAs target genes was generated using the STRING database (version 12.0, https://string-db.org/, accessed on 6 November 2023). The medium confidence was set to 0.4. Then, the PPI network was visualized in Cytoscape (version 3.10.1) and degree values were calculated using the CytoNCA plug-in (version 2.1.6) to identify important nodes in the network. The cytoHubba plug-in (version 0.1) was used to calculate hub genes from the PPI network, and the top 20 genes were filtered out using the method of degree.

### 2.9. Validation

In order to confirm the small RNA sequencing results, DE-miRNAs were randomly selected for qRT-PCR validation. Total RNA was extracted using TRIzol™ Reagent (Invitrogen, Carlsbad, CA, USA), and 2 μg of total RNA was reverse-transcribed to cDNA using the miRNA 1st Strand cDNA Synthesis Kit (Vazyme, Nanjing, China) according to the manufacturer’s instructions. The qRT-PCR reaction was performed using the miRNA Universal SYBR qPCR Master Mix (Vazyme, Nanjing, China) with a real-time PCR system (Bio-Rad, Hercules, CA, USA). U6 was utilized as an endogenous internal control. The primers were synthesized and are listed in Table S1.

### 2.10. Statistical Analysis

Data were presented as mean ± standard deviation (Mean ± SD). Student’s *t*-test was used for the data analysis using GraphPad Prism software (version 9.0). *p* < 0.05 was considered to be statistically significant.

## 3. Results

### 3.1. Ferroptosis Cell Model Establishment

**Figure 2:**
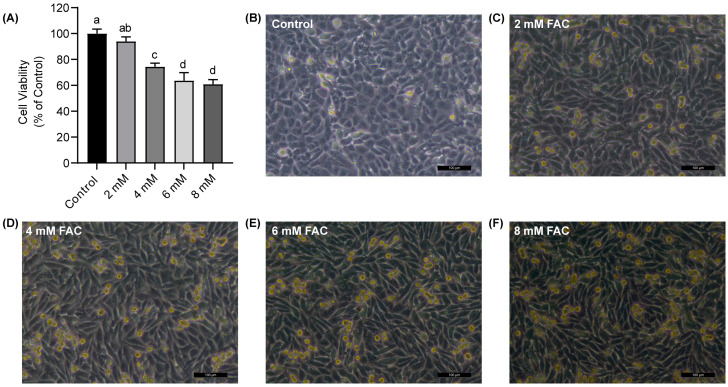
Ferroptosis cell model establishment. (**A**) Cell viability of HT-1080 cells treated with ferric ammonium citrate (FAC) for 24 h using CCK-8 assay. (**B**–**F**) Morphology of HT-1080 cells treated with FAC under different concentrations (0, 2, 4, 6, and 8 mM) for 24 h, scale bar = 100 μm. Data are presented as mean ± SD (*n* = 6). ^a, b, c, d^ Values of the bars without a common letter differ significantly at *p* < 0.05.

After treating HT-1080 cells with varying concentrations of FAC for 24 h, cell viability was assessed using the CCK-8 assay. The results revealed that FAC affected the survival of the HT-1080 cells in a dose-dependent manner (Figure 2A). When the FAC concentration reached 4 mM, the cell viability decreased significantly to about 75% (*p* < 0.05). Next, an inverted microscope was used to observe the morphology of cells. We observed wrinkling and shedding in certain cell morphology, along with cell death following iron treatment (Figure 2B–F). These findings were consistent with the validation results of the FAC-induced ferroptosis model previously established, suggesting the successful establishment of a ferroptosis model in HT-1080 cells.

### 3.2. Overview of Small RNA Sequencing

To investigate the changes in miRNAs expression profile following ferroptosis in HT-1080 cells, six small RNA libraries were constructed from the control group (CO1, CO2, and CO3) and the iron group (IO1, IO2, and IO3). As displayed in Table 1, the number of raw reads per sample after high-throughput sequencing ranged from 11,236,010 to 14,708,693 reads. Further, the data were filtered to obtain clean reads, with 11,066,190–14,490,295 reads per sample. Finally, 1346 known matured miRNAs and 80 novel miRNAs were detected after filtering and matching.

### 3.3. miRNA Differential Expression Analysis

**Figure 3:**
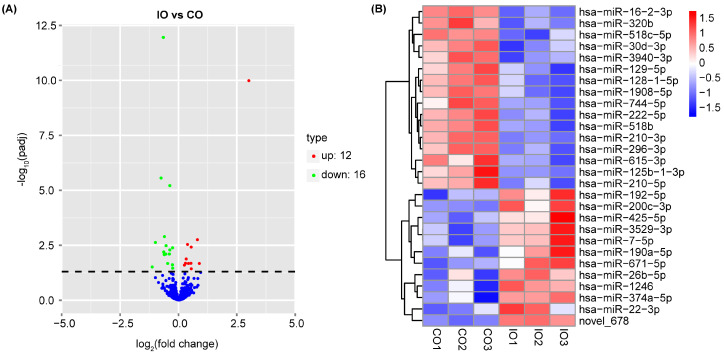
Differentially expressed miRNAs (DE-miRNAs) between the control group (CO) and iron group (IO). (**A**) Volcano plot. Red color represents significantly up-regulated miRNAs, and green color represents significantly down-regulated miRNAs (*p* adj  < 0.05). Blue color represents miRNAs with no significance. (**B**) Hierarchical clustering analysis of relatively high expression miRNAs (red) and relatively low expression miRNAs (blue).

As shown in the volcano plot (Figure 3A), the expression pattern in the control group and the iron group was clearly distinguished. A total of 12 genes were significantly up-regulated, and 16 genes were down-regulated in the iron group compared to the control group. Further, we plotted the differential miRNAs that met the differential screening criteria into a heat map using hierarchical clustering analysis (Figure 3B). Novel_678, hsa-miR-200c-3p, and hsa-miR-192-5p turned to be the most prominent significantly up-regulated DE-miRNAs (*p* adj < 0.05). hsa-miR-1908-5p, hsa-miR-128-1-5p, and hsa-miR-296-3p were the most prominent significantly down-regulated DE-miRNAs (*p* adj < 0.05).

### 3.4. Validation of DE-miRNAs

**Figure 4:**
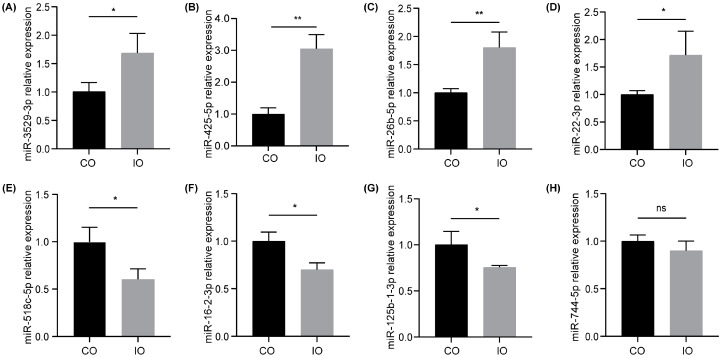
Validation of DE-miRNAs in HT-1080 cells between the control group (CO) and the iron group (IO) using qRT-PCR. (**A**–**D**) Up-regulated DE-miRNAs: miR-3529-3p (**A**), miR-425-5p (**B**), miR-26b-5p (**C**), and miR-22-3p (**D**). (**E**–**H**) Down-regulated DE-miRNAs: miR-518c-5p (**E**), miR-16-2-3p (**F**), miR-125b-1-3p (**G**), and miR-744-5p (**H**). Data are represented as the mean ± SD (*n* = 3), * *p*  < 0.05, ** *p*  < 0.01; ns, not significant.

We randomly selected four up-regulated DE-miRNAs (has-miR-3529-3p, has-miR-425-5p, has-miR-26b-5p, and has-miR-22-3p) and four down-regulated DE-miRNAs (has-miR-518c-5p, has-miR-16-2-3p, has-miR-125b-1-3p, and has-miR-744-5p) to verify the validity of the small RNA sequencing data using qRT-PCR. According to the results of qRT-PCR, most of the selected DE-miRNAs exhibited significant differences, consistent with the trend of the sequencing results (Figure 4A–G, *p* < 0.05). hsa-miR-744-5p presented a lower tendency without a statistical difference (Figure 4H, *p* > 0.05). Overall, the qRT-PCR results confirmed the reliability of the small RNA sequencing data.

### 3.5. Prediction of TFs of DE-miRNAs

**Figure 5:**
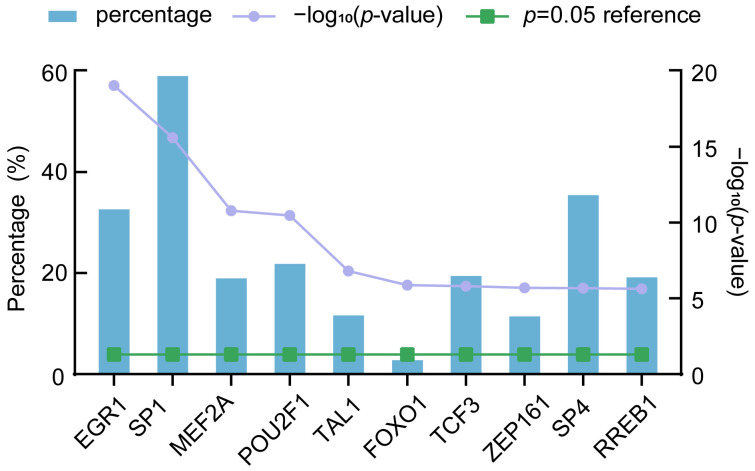
Predicted transcription factors (TFs) of DE-miRNAs. EGR1—early growth response 1; FOXO1—forkhead box O1; MEF2A—myocyte enhancer factor 2A; POU2F1—POU class 2 homeobox 1; RREB1—Ras-responsive element binding protein 1, SP1—Sp1 transcription factor; SP4—Sp4 transcription factor; TAL1—basic helix-loop-helix (bHLH) transcription factor 1; TCF3—transcription factor 3; and ZFP161—zinc finger protein 161.

We predicted the TFs of DE-miRNAs using FunRich. The top 10 TFs were filtered out, including EGR1 (33.50%), SP1 (59.90%), MEF2A (19.80%), POU2F1 (22.70%), TAL1 (12.50%), FOXO1 (3.60%), TCF3 (20.30%), ZFP161 (12.30%), SP4 (36.30%), and RREB1 (20%) (Figure 5). The results indicated that the top 10 TFs had regulatory relationships with DE-miRNAs, and SP1 was found to regulate most DE-miRNAs.

### 3.6. Prediction of Target Genes of DE-miRNAs

**Figure 6:**
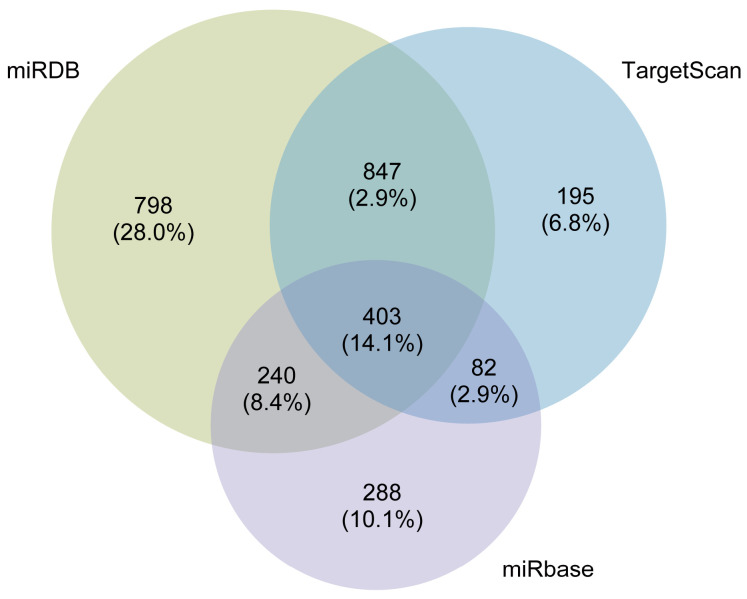
The Venn diagram of the predicted target genes using miRDB, TargetScan, and miRBase databases.

The miDIP online platform was utilized to predict the downstream target genes of DE-miRNAs. As shown in Figure 6, 2288, 1013, and 1527 target genes were predicted from miRDB, miRbase, and TargetScan databases, respectively. Then, we screened a total of 403 target genes overlapping across the three databases for subsequent analysis.

### 3.7. GO and KEGG Enrichment Analyses

**Figure 7:**
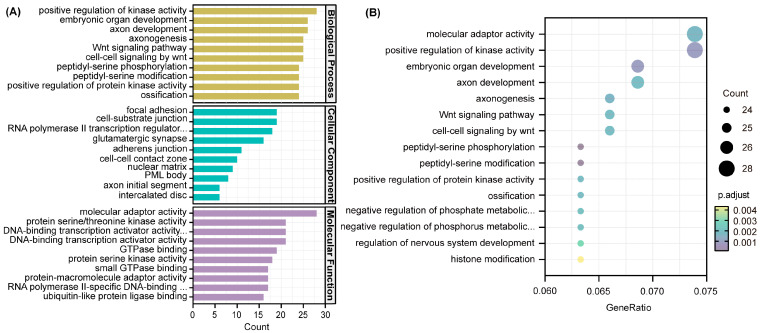
Gene ontology (GO) enrichment analysis of target genes. (**A**) Bar plot. (**B**) Scatter plot. The diameter of a point represents the number of target genes enriched in a specific item, and the adjusted *p*-value represents the degree of enrichment.

GO and KEGG analyses were used to investigate the key function and pathway of target genes of DE-miRNAs using the clusterProfiler package in R. Figure 7A shows the top 10 most numerous items among the categories of biological process, cell component, and molecular function, respectively. GO results showed that the most abundant target genes were enriched in biological process. Then, we selected the top 15 items with the highest enrichment, mainly involved in molecular adaptor activity, positive regulation of kinase activity, embryonic organ development, axon development, axonogenesis, Wnt signaling pathway, and cell–cell signaling by wnt (Figure 7B).

**Figure 8:**
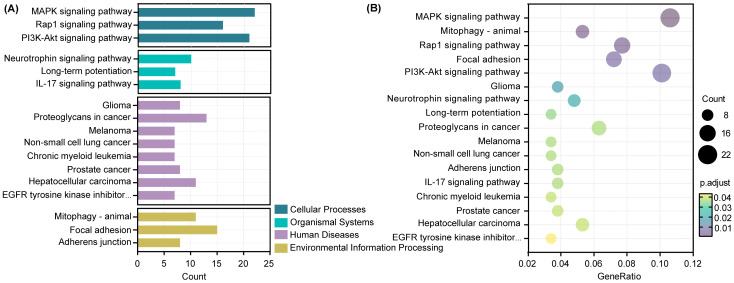
Kyoto Encyclopedia of Genes and Genomes (KEGG) enrichment analysis of target genes. (**A**) Bar plot. (**B**) Scatter plot. The diameter of a point represents the number of target genes enriched in a specific item, and the adjusted *p*-value represents the degree of enrichment.

As shown in Figure 8A, a total of 17 pathways were identified using KEGG analysis, among which human diseases were the pathway with the largest number of target genes. KEGG analysis revealed that target genes were significantly enriched in the MAPK signaling pathway, PI3K-Akt signaling pathway, Rap1 signaling pathway, focal adhesion, and proteoglycans in cancer (Figure 8B).

### 3.8. Construction of Target Genes PPI Network and Hub Genes Network

**Figure 9:**
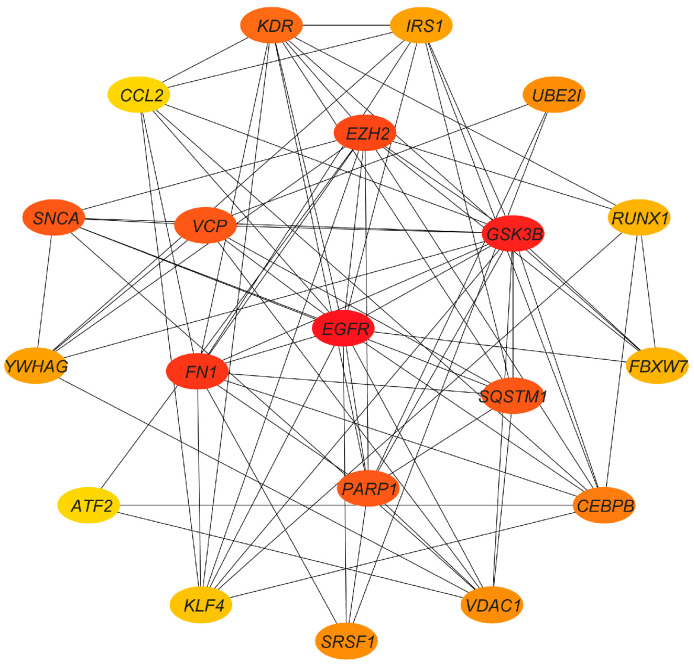
PPI network of hub genes. Red indicates a higher degree of nodes in the PPI network.

The PPI network of target genes was established via the STRING database with medium confidence (0.4) and visualized using Cytoscape. As shown in Figure S1, there were 262 nodes and 1486 edges in the PPI network. Then, we screened the top 20 hub genes by degree order using the cytoHubba plug-in and generated the PPI network (Figure 9). The top 10 hub genes were *EGFR*, *GSK3B*, *FN1*, *EZH2*, *PARP1*, *VCP*, *SNCA*, *SQSTM1*, *KDR*, and *CEBPB*.

### 3.9. Construction of the DE-miRNAs-Target Genes Regulatory Network

**Figure 10:**
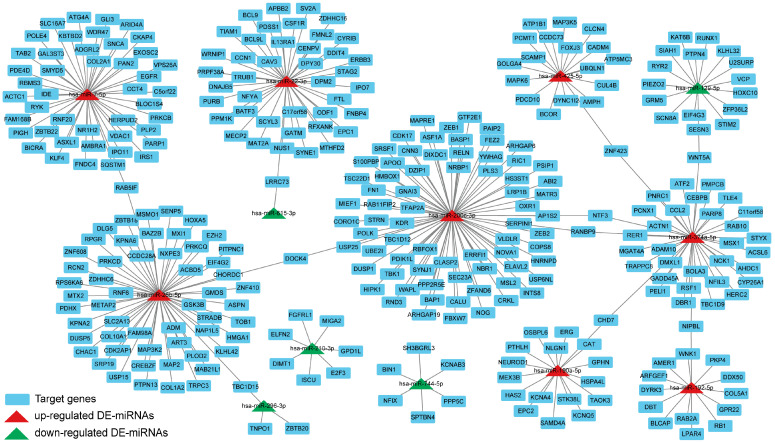
Network of interaction between DE-miRNAs and target genes via Cytoscape. Red color represents up-regulated DE-miRNAs, green color represents down-regulated DE-miRNAs, and blue color represents target genes.

To investigate the key DE-miRNAs, we constructed the DE-miRNAs-target genes regulatory network. As shown in Figure 10, there were eight up-regulated DE-miRNAs, five down-regulated DE-miRNAs, and 337 target genes with 340 nodes and 337 edges in the regulatory network. Topological analysis of the network using the CytoNCA plug-in revealed that key up-regulated miRNAs with high degree included hsa-miR-200c-3p, hsa-miR-26b-5p, and hsa-miR-7-5p, and key down-regulated miRNAs with high degree included hsa-miR-210-3p, hsa-miR-744-5p, and hsa-miR-296-3p.

## 4. Discussion

Iron is a fundamental micronutrient, which plays a crucial role in the life activities of organisms. Small RNA can influence various cellular pathways by acting as regulators of gene expression at the translational and transcriptional levels and play an indispensable role in cell proliferation and differentiation, transposon regulation, genome stabilization, and other life activities [21,22]. Among them, miRNAs can affect the progression of ferroptosis through different pathways [13]. In our previous study, we demonstrated that FAC can induce ferroptosis in HT-1080 cells, which are sensitive to ferroptosis [18]. However, the role of miRNAs in ferroptosis in HT-1080 cells remains unknown. Thus, we aimed to identify the DE-miRNAs, upstream TFs, upstream target genes, hub genes, and regulatory networks of ferroptosis in HT-1080 cells based on small RNA sequencing and bioinformatics analysis.

In this study, we established a model of ferroptosis induced by FAC-induced intracellular iron overload in HT-1080 cells. Cell viability is an essential indicator for evaluating iron toxicity. Considering the post-transcriptional expression level and the quality of RNA extraction, we selected a treatment dose of about 75% cell viability for subsequent RNA sequencing. miRNAs expression profile was obtained, including 1346 known matured miRNAs and 80 matured novel miRNAs. DE-miRNAs were further screened, and 12 miRNAs were found to be significantly up-regulated. Novel-678 was the predicted novel miRNA with the most significant fold change of up-regulated DE-miRNAs, but previous studies are lacking. Except for novel-678, miR-1246 underwent the most significant up-regulation. A previous study has found that p53 up-regulated miR-1246 expression and induced apoptosis [23], which may explain the reduced cell viability at the miRNA level. Cellular iron level is mainly regulated by the iron-responsive element-iron-regulatory protein (IRE-IRP) system, which maintains the balance of iron transport, storage, and utilization [2]. miR-7-5p can target 3′-TfR1 IREs and down-regulate the expression of TfR1, thereby alleviating the uptake of exogenous iron sources [24]. In addition, it has been confirmed that the knockdown of miR-7-5p can lead to the up-regulation of the ferroptosis marker *ALOX12* gene, thereby enhancing lipid peroxidation [25]. These results suggest that miR-7-5p influences ferroptosis by regulating iron metabolism and lipid metabolism. In our study, miR-7-5p was significantly up-regulated, which may be related to self-regulation after ferroptosis. A logistic regression model developed from a population-based health study reflected a positive correlation between iron overload and impaired glucose metabolism [26]. The glucose-intolerant population had increased miR-26b-5p expression and exhibited high serum ferritin levels compared to the normal group [27]. The significant up-regulation of miR-26b-5p reflected glucose metabolism abnormalities after ferroptosis. The exosome miR-22-3p from bone marrow mesenchymal stem cells inhibited colorectal cancer cells proliferation and invasion through the PI3K/AKT pathway [28]. The up-regulation of miR-22-3p expression by iron overload may restrict cell proliferation and migration and induce cell apoptosis. Meanwhile, two members of the miR-210 family (miR-210-3p and miR-210-5p) were found to be down-regulated. miR-210 could work as an iron sensor and participate in the maintenance of iron homeostasis by sustaining the targets expression level [29]. miR-1908-5p was the down-regulated DE-miRNA with the highest fold change. A previous study has reported that miR-1908-5p was involved in metabolic and energy regulation through multiple independent pathways in hepatocytes [30]. miR-128-1-5p expression was reduced in mice following myocardial ischemia/reperfusion injury and negatively regulated its direct target Gadd45g, leading to cardiomyocyte apoptosis [31]. Therefore, we may have to be alert to the possibility that the down-regulation of miR-128-1-5p after iron overload may promote apoptosis. In addition, reports on other DE-miRNAs related to iron metabolism or ferroptosis are limited and need to be further explored in the future. 

The expression of miRNAs is regulated by the level of transcription, which is mediated by the epigenetic control of TFs and DNA methylation [32]. TFs are important gene regulators, which can be used as the upstream regulators of miRNAs to activate or inhibit miRNAs expression [33]. There is increasing evidence that abnormal regulation of miRNA by TFs can lead to diseases [34]. TFs and miRNAs alter the expression of each other, and it has been suggested that positive and negative transcriptional co-regulatory loops of miRNAs and their targets are common in mammalian systems [35]. We used the Funrich software to predict the TFs that might regulate DE-miRNAs, primarily EGR1, SP1, and MEF2A. Ferroptosis has vital effects on the development of osteoarthritis, and *EGR1* has been identified as one of the possible diagnostic biomarkers based on bioinformatics analysis of ferroptosis-related genes [36]. SP1 is a promoter-specific binding factor that has been shown to be overexpressed in numerous cancers and is associated with poor prognosis [37]. MEF2A is important for cell proliferation, differentiation, and survival [38]. A previous study has demonstrated that the activation of HIF-1A-FOX3 and MEF2A pathways can induce apoptosis in hepatocellular carcinoma cells [39]. In addition, FOXO1 is worthy of attention as it plays a key functional role as a tumor suppressor in a variety of cancers and is associated with different types of cancers [40].

Genes are not completely independent and interact with other genes. In the case of miRNAs, they usually play a regulatory role by regulating the expression of target genes to affect downstream molecules. We further used the miRDB, miRbase, and TargetScan databases to predict the downstream target genes and identified the overlapping target genes. The target genes obtained were analyzed using GO annotation to elucidate the effect of ferroptosis on function. The majority of entries were enriched in biological process and mainly involved in the positive regulation of kinase activity, embryonic organ development, and axon development. We further analyzed the signaling pathways of the target genes using KEGG, and the main enriched pathways were the MAPK signaling pathway, Rap1 signaling pathway, and PI3K-Akt signaling pathway. AMPK, a sensor of cellular energy status, affects ferroptosis sensitivity by inhibiting the activity of acetyl-CoA carboxylase, which is required for PUFA synthesis [41]. Previous studies have shown that FAC treatment can induce hepatocyte apoptosis through ROS-activated p38 MAPK and NF-κB pathways [42]. Genes in the PI3K-Akt pathway are most commonly altered in human cancers, and the aberrant activation of this pathway has been associated with cellular transformation, tumorigenesis, cancer progression, and drug resistance [43]. Additionally, KEGG analysis revealed enrichment in various specific cancer pathways, including hepatocellular carcinoma, glioma, prostate cancer, melanoma, non-small cell lung cancer, and chronic myeloid leukemia. A growing body of evidence suggests that ferroptosis, as a unique cell death mechanism, is a crucial strategy in cancer treatment [44]. The unique metabolism of cancer cells, high ROS load, and specific mutations make cells more susceptible to ferroptosis, exposing vulnerabilities in certain types of cancer that can be targeted for treatment [8], such as hepatocellular carcinoma, prostate cancer, and fibrosarcoma [45,46].

The screened target genes were used to construct PPI network, and topological heterogeneity was used to screen hub genes. Meanwhile, we compared the obtained hub genes with the ferroptosis database (FerrDb V2) [47]. The hub genes *EGFR*, *GSK3B*, and *SNCA* function as drivers promoting ferroptosis, while *PARP1* and *VCP* act as suppressors impeding this process. Within human mammary epithelial cells, the absence of cystine induces ferroptosis, particularly in cells expressing activated *EGFR* mutants [48]. Simultaneously, *EGFR* has the capacity to engage with ligands, forming either homodimers or heterodimers, thereby activating PI3K signaling pathways [49]. *GSK3B* encodes a multifunctional serine/threonine protein kinase and serves as a pivotal downstream factor within the PI3K-Akt pathway [50]. *PARP-1* encodes a DNA damage sensor protein that is involved in DNA repair and various cellular processes, holding significant clinical potential [51]. *VCP* encodes the VCP protein, which is a novel regulator of autophagy initiation and is involved in ferroptosis [52]. Finally, we constructed the PPI network between DE-miRNAs and target genes and found that the up-regulated DE-miRNAs had more nodes, including hsa-miR-200c-3p, hsa-miR-26b-5p, and hsa-miR-7-5p. This indicates that up-regulated DE-miRNAs may play a more critical role in ferroptosis.

## 5. Conclusions

In summary, ferroptosis alters the miRNAs expression profile of HT-1080 cells and implicates various biological processes and signaling pathways, where hsa-miR-200c-3p, hsa-miR-26b-5p, and hsa-miR-7-5p may play pivotal roles. Our findings amplify the changes in miRNAs under iron overload and provide new potential targets for ferroptosis-targeted therapy for cancer.

## Figures and Tables

**Table 1:** Statistical analysis of the small RNA libraries.

Sample	Raw Reads	Clean Reads	Total Reads	Mapped Reads	Matured miRNA	
					Known miRNA	Novel miRNA
CO1	14,708,693	14,490,295	12,890,534	11,542,419	1012	53
CO2	13,213,177	12,863,314	10,856,522	9,830,156	996	53
CO3	12,471,423	12,123,043	10,138,113	9,126,551	998	53
IO1	11,236,010	11,066,190	9,047,944	7,702,918	958	50
IO2	13,314,751	13,114,956	10,692,894	9,134,142	966	52
IO3	12,246,380	11,905,219	10,680,970	8,885,369	956	48

## Data Availability

The data presented in this study are available on request from the corresponding author due to privacy.

## Supplementary Materials

The following supporting information can be downloaded at https://www.mdpi.com/article/10.3390/nu16060873/s1. Table S1: miRNA primers used for qRT-PCR; Figure S1: Protein–protein interactions (PPI) network of target genes. The size and color indicate the degree of node in the PPI network.

## Abbreviations

DE-miRNA	differentially expressed miRNA
FAC	ferric ammonium citrate
GO	Gene ontology
KEGG	Kyoto Encyclopedia of Genes and Genomes
miRNA	microRNA
PPI	protein–protein interaction
ROS	reactive oxygen species
TF	transcription factor
