# Supplementary material for: Ferroptosis Altered microRNAs Expression in HT-1080 Fibrosarcoma Cells Based on Small RNA Sequencing and Bioinformatics Analysis
Source: Nutrients. 2024 Mar 17;16(6):873. doi: 10.3390/nu16060873 (PMC10976119; doi:10.3390/nu16060873)
Supplement: Supplementary file 1 [file nutrients-16-00873-s001.zip › nutrients-2858558-supplementary.pdf]

Table S1. miRNA primers used for qRT-PCR.

miRNA	Sequence
hsa-miR-3529-3p	F: AGTGCAGGGTCCGAGGTAT<br>R: AACAACAAAATCACTAGTCTTCCAG
hsa-miR-425-5p	F: AGTGCAGGGTCCGAGGTAT<br>R: AATGACACGATCACTCCCGTT
hsa-miR-26b-5p	F: AGTGCAGGGTCCGAGGTAT<br>R: TTCAAGTAATTCAGGATAGGTGTCG
hsa-miR-22-3p	F: AGTGCAGGGTCCGAGGTAT<br>R: TGAAGAACTGTGTCGTATCCA
hsa-miR-518c-5p	F: AGTGCAGGGTCCGAGGTAT<br>R: TCTCTGGAGGGAAGCACTTTC
hsa-miR-16-2-3p	F: AGTGCAGGGTCCGAGGTAT<br>R: CCAATATTACTGTGCTGCTTTAG
hsa-miR-125b-1-3p	F: TAATACTGCCGGGTAATGATGGA<br>R: ACGGGTTAGGCTCTTGGGA
hsa-miR-3529-3p	F: AGTGCAGGGTCCGAGGTAT<br>R: AACAACAAAATCACTAGTCTTCCAG
hsa-miR-425-5p	F: AGTGCAGGGTCCGAGGTAT<br>R: AATGACACGATCACTCCCGTT
hsa-miR-26b-5p	F: AGTGCAGGGTCCGAGGTAT<br>R: TTCAAGTAATTCAGGATAGGTGTCG
hsa-miR-22-3p	F: AGTGCAGGGTCCGAGGTAT<br>R: TGAAGAACTGTGTCGTATCCA
hsa-miR-518c-5p	F: AGTGCAGGGTCCGAGGTAT<br>R: TCTCTGGAGGGAAGCACTTTC
hsa-miR-16-2-3p	F: AGTGCAGGGTCCGAGGTAT<br>R: CCAATATTACTGTGCTGCTTTAG
hsa-miR-125b-1-3p	F: TAATACTGCCGGGTAATGATGGA<br>R: ACGGGTTAGGCTCTTGGGA
hsa-miR-744-5p	F: AGTGCAGGGTCCGAGGTAT<br>R: GGCTAGGGCTAACAGCAGTC
U6	F: GCTTCGGCAGCACATATACTA<br>R: GGAACGCTTCACGAATTTGC

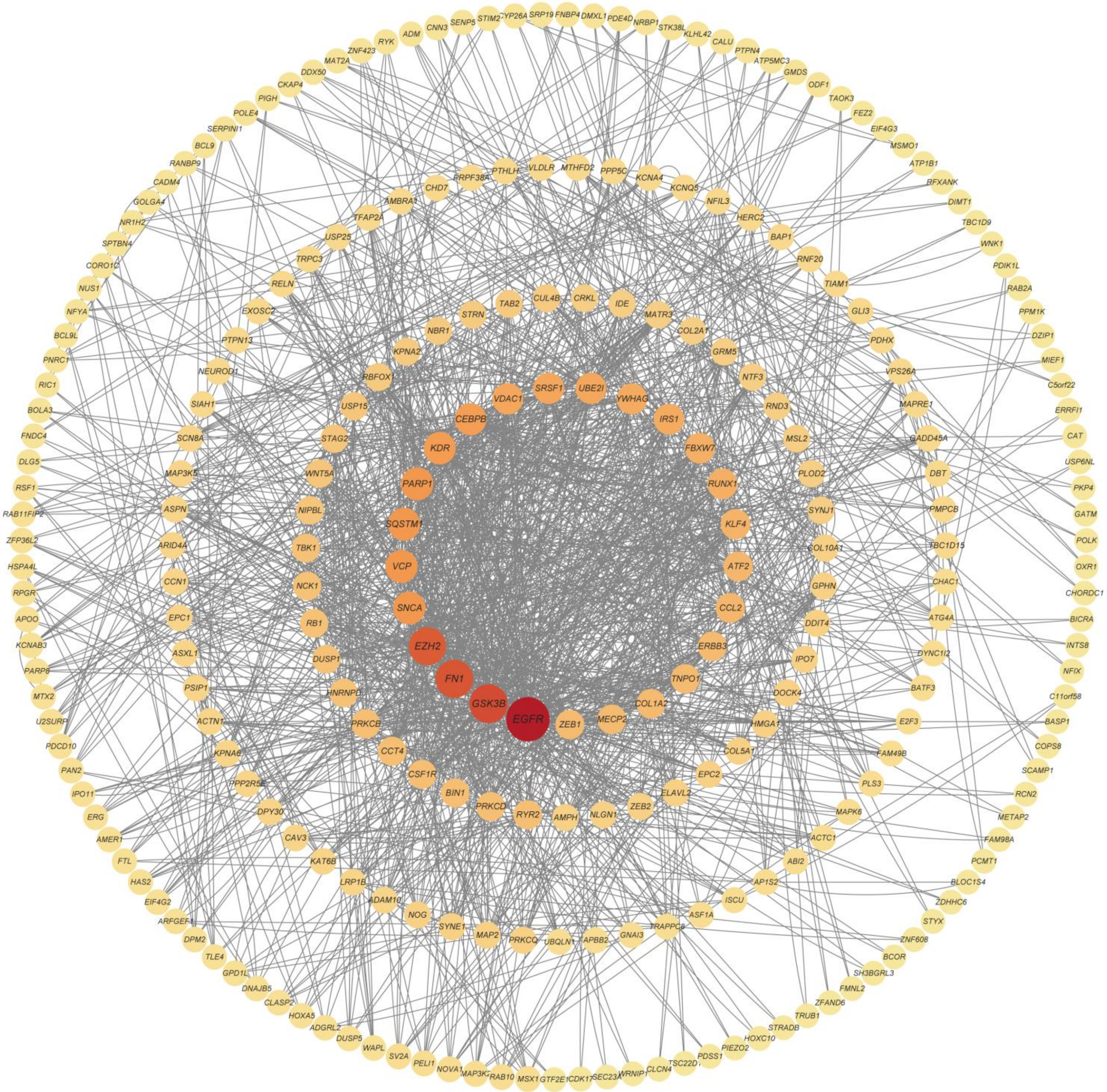

Figure S1. Protein–protein interactions (PPI) network of target genes. The size and color indicated the degree of node in the PPI network.
